# Designing a green poly(β-amino ester) for the delivery of nicotinamide drugs with biological activities and conducting a DFT investigation

**DOI:** 10.1039/d3ra08585f

**Published:** 2024-02-13

**Authors:** M. S. Hashem, Asmaa M. Fahim, F. M. Helaly

**Affiliations:** a Polymers and Pigments Department, National Research Centre Dokki, P.O. Box. 12622 Giza Egypt ms.hashem@nrc.sci.eg; b Department of Green Chemistry, National Research Centre Dokki, P.O. Box. 12622 Giza Egypt asmaamahmoud8521@gmail.com

## Abstract

The environmentally friendly polymerization process was carried out using microwave irradiation without additional solvents or catalysts to produce poly(β-amino ester) (PβAE) which served as a drug delivery system. PβAE was synthesized through Michael addition polymerization of 1,4-butane diol diacrylate and piperazine. Swelling and biodegradation studies were conducted in various solvents and phosphate-buffered saline (PBS, pH 7.4) at 37 °C to evaluate the properties of the polymeric gel. The PβAE matrix demonstrated solubility enhancement for hydrophobic antimicrobial and antitumor-active nicotinamide derivatives (TEINH, APTAT, and MOAPM), controlling their release over 10 days in (PBS). The successful formation of free and loaded PβAE with nicotinamide active materials was confirmed by spectroscopic analysis including Fourier-transform infrared spectroscopy (FT-IR) and scanning electron microscopy (SEM). Optimization and physical descriptor determination *via* the DFT/B3LYP-631(G) basis set were performed to aid in the biological evaluation of these compounds with elucidation of their physical and chemical interaction between poly(β-amino ester) and nicotinamide drugs.

## Abstract 2

Poly(β-amino ester) was synthesized through addition polymerization under microwave irradiation, demonstrating antimicrobial and anticancer activities against MCF-7 tumor cells, along with an impressive ability to prevent drug leakage.

## Graphical abstract

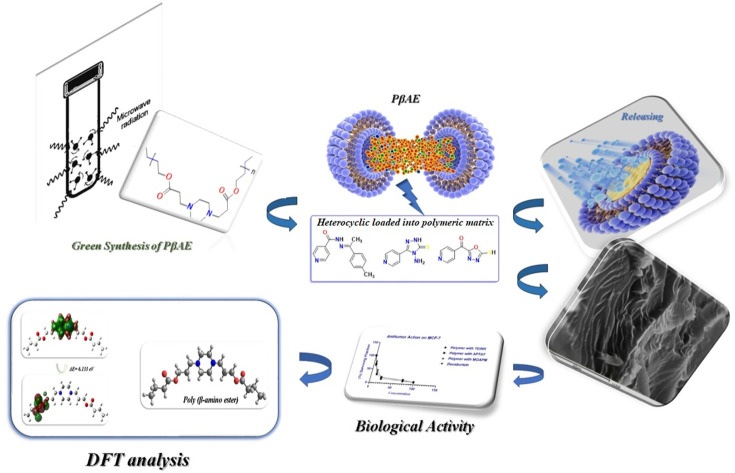

## 1. Introduction

Chemotherapy is the cornerstone of cancer treatment because it reduces discomfort and prevents the spread of cancer cells.^1^ However, chemotherapeutic drugs with small molecules tend to lack selectivity and diffuse strongly, leading to a variety of undesirable effects and usage limitations.^2,3^ Recent advancements in vehicular substances have provided an opportunity for chemotherapeutic agents to control their dispersion and selectivity in the body, thereby reducing their harmful complications.^4–6^ Given their unique properties, numerous polymers have been identified as potential pharmaceutical delivery systems for chemotherapy drugs, which often have significant side effects.^7–12^ Biodegradable polymers have proven to be essential in various medical and related applications. To meet the demands of drug delivery that require biodegradable polymers, several newly fabricated biodegradable polymers and natural polymeric materials have been developed in recent decades.^13–16^

With a polymer matrix system (a matrix that is insoluble in water) or a reservoir system (a polymeric membrane that is soluble in water), gels function as regulated drug delivery vehicles through the diffusion process. Hydrogels, aerogels, and organ gels are used in pharmaceuticals as methods regulated or sustained drug delivery. Owing to their suppleness, elasticity, and reduced interfacial tension in biological and aqueous conditions, these polymeric materials are like natural tissues. Moreover, their exceptional gel ability, biodegradability, and compatibility with living tissues, have made them widely used in the medical sectors.^17–19^

**Fig. 1:**
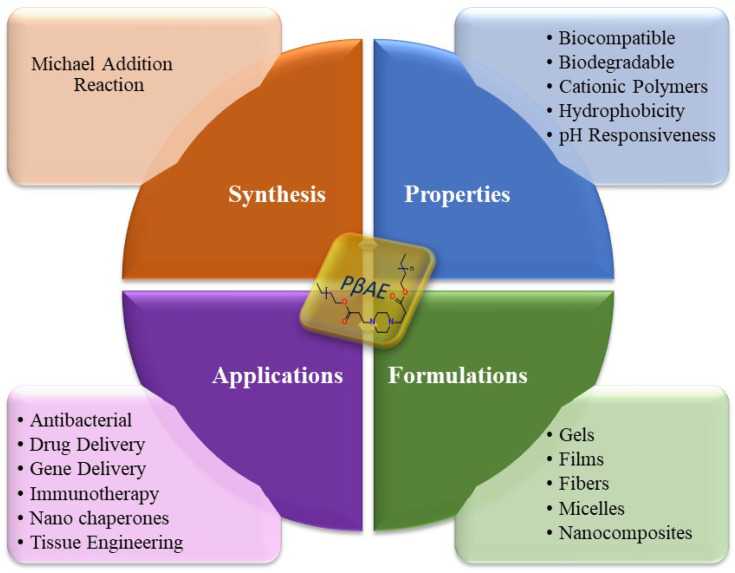
PβAE: synthesis, properties, formulations, and applications.

Poly(β-amino ester) (PβAE), a category of biodegradable cationic polymer, was initially developed for gene delivery through a Michael addition process. It possesses unique properties that make it suitable for controlling drug release in pharmaceutical and biomedical applications, as displayed in Fig. 1. PβAE is an ideal carrier for delivering anticancer drugs because of its distinct characteristics including biocompatibility, low cytotoxicity, pH-responsiveness, and ease of production.^20–22^ At physiological pH, PβAE is neutrally hydrophobic, but under acidic conditions, it becomes positively charged and hydrophilic when protonated. This allows for chemical functionalization and the ability to adjust its structure. By using PβAE as the hydrophobic block of an amphiphilic copolymer, its self-assembled structure can be disrupted in an acidic environment, transforming it into a hydrophilic component. Typically, the polymer is synthesized by combining acrylates and amines with a solvent at high temperatures for several hours.^23–25^ However, the use of solvents presents challenges due to their reported cytotoxic activities. Therefore, an alternative method utilizing environmentally safe microwave radiation was explored for an easy and quick preparation process.

In this study, the eco-friendly formation of the PβAE matrix was achieved without using any solvents or catalysts *via* microwave irradiation and characterized through spectral investigation.^26^ Moreover, this polymer was used as a delivery system for different heterocyclic active antitumor materials.^27^ The release rate of the active components was analyzed to determine how well the produced polymer holds the medicine for controlled release. The poly(β-amino ester) and the adsorbed heterocycles exhibited antimicrobial and antitumor activity on MCF-7 tumor cell, which can be released from the surface of the polymer. In addition, the released pharmaceutical materials from PβAE will undergo bioassay measurements against an antimicrobial and human breast cancer cell line. Furthermore, the physical descriptors of the poly(β-amino ester) and the isonicotinic heterocycle derivatives utilized in the DFT/B3LYP/6-31(G) basis set showed that their addition to the surface of the polymer enhanced their activity with physical and hydrogen bond interaction, which correlated with experimental analysis and investigated their HOMO–LUMO band energy gap.^28^

## 2. Experimental section

### 2.1. General procedure

The synthesized polymer was subjected to various evaluation methods: FT-IR spectra were obtained using KBr pellets on an FT-IR spectrometer (Nicolet 670, range: 4000 to 400 cm^−1^, USA). Surface morphology was analyzed using a scanning electron microscope (JXA-840A Electron probe microanalyzer, JEOL, Japan) with an accelerating voltage of 30 kV after coating with a gold film *via* the S150A Sputter Coater (Edwards, England). Drug loading and release investigations were conducted using a double-beam spectrophotometer (Shimadzu UV-2401 PC, Japan). Microwave experiments utilized the CEM Discovering LabMate microwave device (300 W, ChemDriver software; Matthews, NC) in a sealed chamber with pressure, employing microwave-irradiated covered-Pyrex tubes.

### 2.2. Materials and reagents

1,4-Butanediol diacrylate (technical grade, contains ∼75 ppm hydroquinone as inhibitor) and piperazine [assay ≥99% (GC)] were obtained from Sigma-Aldrich. Dimethyl formamide (anhydrous, 99.8%) (DMF), dimethyl sulfoxide (anhydrous, ≥99.9%) (DMSO), and tetrahydrofuran (anhydrous, ≥99.9%, inhibitor-free) (THF) were purchased from Aldrich. The nicotinamide derivatives; 7(*E*)-*N*′-(1-(*p*-tolyl)ethylidene)isonicotinohydrazide (TEINH), 4-amino-3-(pyridin-4-yl)-1*H*-1,2,4-triazole-5(4*H*)-thione (APTAT), and (5-mercapto-1,3,4-oxadiazol-2-yl)(pyridin-4-yl)methanone (MOAPM) were previously synthesized and evaluated as promising bioactive anticancer materials.^29^

### 2.3. Synthesis of poly(β-amino ester) gel matrix

The PβAE creation was achieved using 1,4-butanediol diacrylate and piperazine without the need for any solvents. Piperazine crystals and 1,4-butanediol diacrylate were combined in an HP-500 Plus process vessel. The vessel was securely sealed and then microwaved at 110 °C and 17.2 bar of pressure for 10 minutes. This resulted in the formation of a thick and creamy yellow gel which was immersed in ethanol and diethyl ether for one week to eliminate any unreacted monomers and homopolymers. Subsequently, the formulated PβAE was dried under vacuum to ensure the removal of any remaining solvents. The polymerization process was then repeated using different acrylate-to-amine molar ratios, specifically 1 : 1, 1 : 2.3, and 2.3 : 1.

### 2.4. Swelling behavior of poly(β-amino ester) matrix

The expanding features of PβAE were investigated at room temperature using DMSO, DMF, and THF, some of these solvents have been used in previous research.^29^ First, the initial mass of the synthesized PβAE gel was terminated (*W*_i_), and then the gel was immersed in the respective solvents at room temperature. At specific intervals, the polymeric gel was taken out from the solvents, and its enlarged weight (*W*_s_) was measured. The dynamic variation in weight of the PβAE over time was estimated by the subsequent equation:1
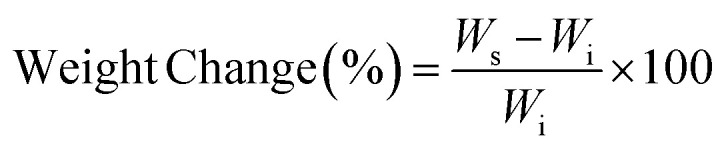
where *W*_i_ and *W*_s_ are the weights of the PβAE in the dry and swollen states, respectively.

### 2.5. *In vitro* degradation of PβAE

The degradation of the PβAE *in vitro* was examined using the loss of mass approach. To generate a PβAE gel, 100 mg of PβAE was placed in 4 ml glass vials at pH 7.4 and incubated for 15 minutes at 37 °C. The vials were then incubated at 37 °C with 3 ml of phosphate-buffered saline (PBS, pH 7.4). Each day, the PBS in the vials was replaced with fresh PBS. Afterward, all the samples were collected and freeze-dried, and the weight of the residue was measured. The weight of the degraded gels was determined by comparing the ratio of lyophilized degraded gels to the original gels using the following equation:2
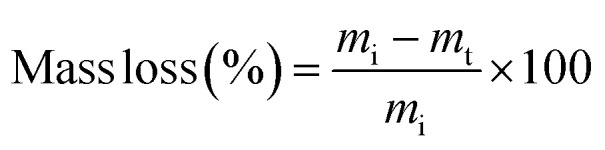
where *m*_i_ is the original mass of the PβAE at the time of polymerization and *m*_t_ is the mass of the PβAE at time *t*.

### 2.6. Loading of antitumor agents in PβAE

The bioactive synthesized therapeutic agents (0.05 g) were combined with the polymeric gel (0.5 g) and the active agent-loaded polymer and equilibrated at room temperature for one day. The drug loading efficiency (DE) was assessed by extracting the drug from the constructed PβAE gels. To achieve full release of the drugs 100 mg of the drug loaded PβAE gels were dispersed in 100 ml of PBS (pH 7.4) and agitated for 30 minutes at 37 °C. The combined mixtures were then diluted to 250 ml with PBS (pH 7.4) and analyzed using a UV-vis's spectrophotometer at wavelengths of 230, 245, and 255 nm after being centrifuged at 4000 rpm for 30 minutes. The DE of the drugs was estimated and described using the following equation:3
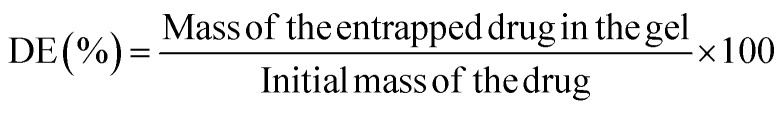


### 2.7. *In vitro* bioactive agents release from PβAE

In a typical approach, polymers loaded with drugs were produced and submerged in 10 ml of PBS (pH 7.4) at 37 °C with horizontal agitation to measure the *in vitro* release of the drugs from the PβAE matrix. At specific intervals, 3 ml of the buffer was removed and replaced with an equal amount of new PBS to maintain the overall volume. The amount of medication released into the medium was calculated by accurately measuring the absorbance at 230, 245, and 255 nm using a UV-vis spectrophotometer in accordance with the calibration curve and represented as follows:4
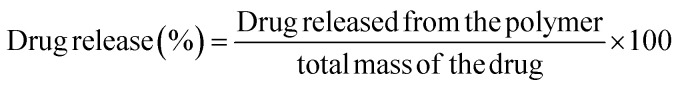


The medicament concentration (mol l^−1^) calculated from the entirety solution volume was the variable used to calculate the amount released drug, and the whole drug was the amount placed in the PβAE gel.

### 2.8. Antimicrobial action

The antimicrobial efficacy of the compounds was assessed through the agar-well diffusion method. *In vitro* antibacterial activity against *Staphylococcus aureus*, *Streptococcus* (Gram-positive bacteria), *Escherichia coli*, *Pseudomonas aeruginosa*, and *Klebsiella pneumonia* (Gram-negative bacteria) was evaluated using nutrient agar medium. The antifungal properties were investigated against *Candida albicans* and *Aspergillus niger* using Sabouraud dextrose agar medium. Ampicillin and Gentamicin served as standard drugs for Gram-positive and Gram-negative bacteria, respectively, while Nystatin was used as a standard drug for fungal strains. DMSO functioned as a negative control solvent. The testing concentration for both bacterial and fungal strains was set at 15 mg ml^−1^.^30–32^ and the dilution method was used from 100 mg ml^−1^ to 0 mg ml^−1^ as a −Ve control.

#### 2.8.1. Method of testing antimicrobial

After applying the sterilizing media (20–25 ml) to each pre-sterilized Petri dish, they were left to solidify at room temperature. To attain a turbidity of OD = 0.13, a microbial suspension was prepared in sterilized saline, equivalent to the McFarland 0.5 standard solution (1.5 × 105 CFU ml^−1^), using a spectrophotometer set at 625 nm. Once the turbidity was adjusted, a sterilized cotton pad soaked in the suspension was placed over the dehydrated agar layer for approximately 15 minutes. Subsequently, the pad was allowed to dry for an additional 15 minutes with the lid on. Using a sterile borer, wells with a diameter of 6 mm were created in the solidified media. Subsequently, 100 μL of the tested compound's solution was added to each well using a micropipette. The plates were then incubated at 37 °C for 24 hours to assess antibacterial activity^33^ The experiment was conducted in triplicate, and the zones of inhibition were measured using a millimeter scale.

#### 2.8.2. Statistical analysis

Statistical analysis was performed to assess differences between samples of the same type of bacteria (or fungi). A one-way analysis of variance (ANOVA) was employed, followed by Duncan's multiple comparisons test using the SPSS package version “22” for Windows. The results are expressed as mean ± standard error (S.E.), and significance levels were set at *p* < 0.05 for statistical significance, *p* < 0.01 for high significance, and *p* < 0.001 for very high significance.

### 2.9. Antitumor activity

The vitality of the cells was evaluated using the MTT (3-[4,5-dimethylthiazol-2-yl]-2,5 diphenyl tetrazolium bromide) assay. This assay relies on the activity of mitochondria, where living cells convert MTT into formazan crystals, allowing the assessment of cytotoxicity.^34^ Using MCF-7 cells, we conducted the MTT assay to evaluate the cytotoxicity of the investigated substances. The cells were seeded at a density of 104 cells per well in 96-well plates and treated with varying concentrations of the test compounds (6.25, 12.5, 25, 50 M) for 48 hours. MTT dissolved in PBS solution was added to each well after 24 hours of incubation at a concentration of 5 mg ml^−1^. The samples were further incubated for 4 hours at 37 °C. Following this, dimethyl sulfoxide (DMSO) was used to dissolve the formazan crystals formed during MTT cleavage in metabolically active cells. The absorbance of formazan in each well was measured at 570 nm using a microplate reader (Model 500; BIORad Instrument Inc., USA). The IC_50_ values of the investigated compounds were estimated based on the relationship between concentrations and mitochondrial activity (%). The negative control received medium instead of the test substance, while doxorubicin (Dox, Mr = 543.5) served as a positive control with 100% inhibition. The testing substance was dissolved in DMSO, with its final concentration in the cells being less than 0.2%. The data is presented as the mean ± SD using Sigma Plot version 12.5, and all experiments were conducted in triplicate.^35^

### 2.10. Computational techniques

We utilized the Gaussian 09W program for our computational calculations, employing the B3LYP hybrid functional (Becke's three-parameter hybrid functional coupled with the B3LYP correlation functional). The geometry optimization was conducted without any symmetrical constraints. This method is known for its accuracy in computing the HOMO–LUMO energy for band gap and physical descriptors within the same basis group size.^9,36^

## 3. Results and discussion

### 3.1. Chemistry

#### 3.1.1. Synthesis of poly(β-amino ester)

**Fig. 2:**
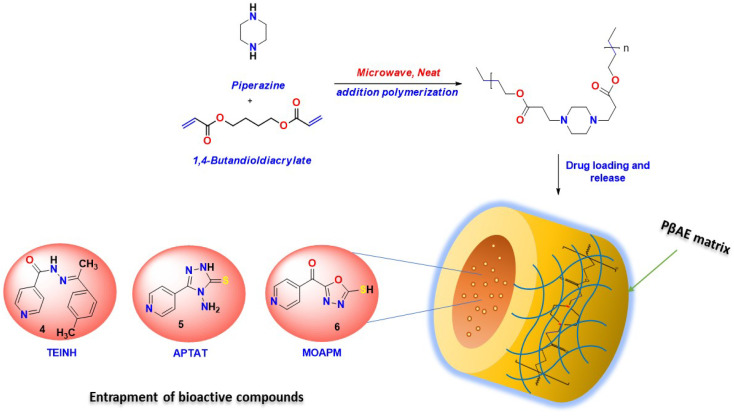
PβAE preparation and loading with pharmaceutical agents.

PβAE with a complex-structure was created through the addition polymerization process of 1,4-butane diol diacrylate and piperazine utilizing microwave irradiation in neat conditions, as shown in Fig. 2. Various molar proportions of the reacting agents were employed to highlight the most suitable regulating product with the best possible features to serve as polymeric pharmaceutical carriers.^37–39^ According to the monomer ratios, Table 1 lists the physical characteristics of the PβAE forms that were produced. With respect to the molar ratio of the reactants, it can be seen *via* a closer look that the products' color shifted from pale yellow to yellow and their texture from gel to crumbly solid. The physical and chemical properties of the generated macromolecule can be changed by varying the molar proportion of the interacting ingredients. In this study, we choose to use the gel form of PβAE for further investigations. To facilitate the retention of the bioactive compounds under study by enhancing the solubility and the capability to release the pharmaceutical materials over a longer period for use as a drug delivery system, PβAE and its entrapped bioactive materials should possess certain properties. FT-IR and SEM spectral evaluations were utilized to analyze the produced polymer before and after pharmaceutical agent loading. The desired bioactive materials were entrapped within the PβAE through physical interaction.

**Table 1:** The molar ratio of the reacting monomers and the physical form of the synthesized PβAE

No.	Reacting monomers (mole)		Mole ratio	Physical forms of PβAE	Color
	1,4-Butanediol diacrylate	Piperazine			
1	0.5	0.5	1 : 1	Gel	Pale yellow
2	0.3	0.7	1 : 2.3	Glutinous solution	Pale yellow
3	0.7	0.3	2.3 : 1	Crumbled solid	Yellow

### 3.2. FT-IR investigation

**Fig. 3:**
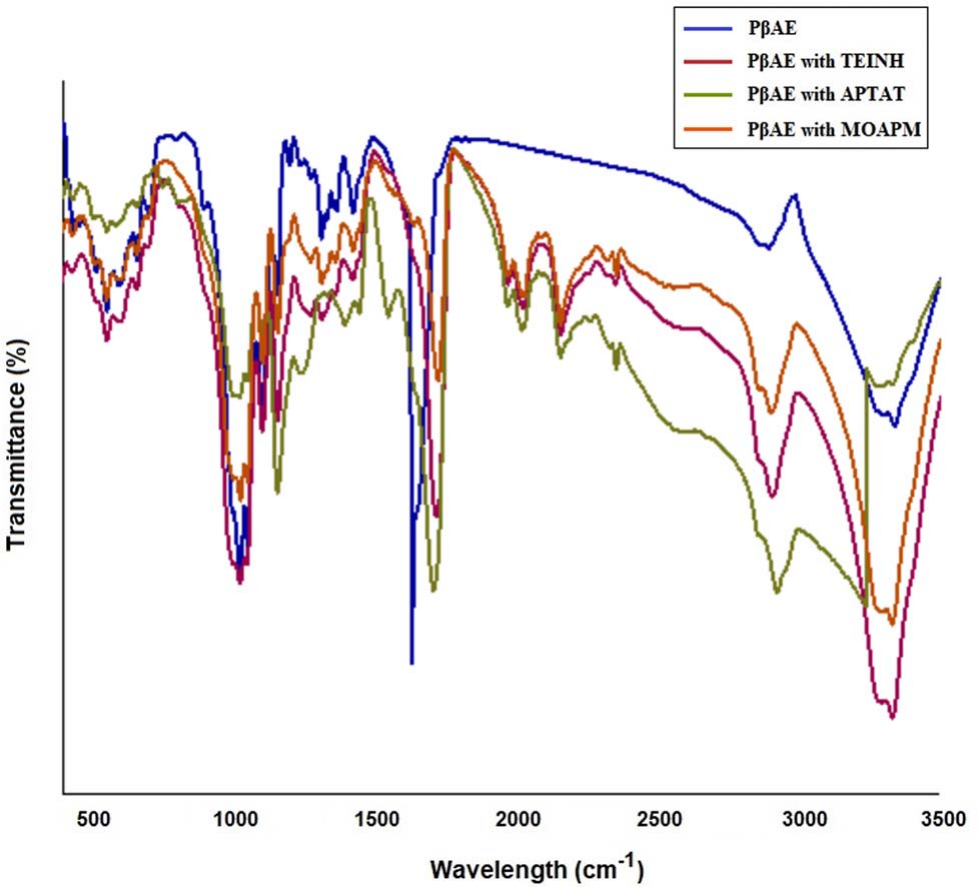
FT-IR of free PβAE and loaded PβAE with TEINH, APTAT, and MOAPM.

Fig. 3 indicates the FT-IR investigation of the synthesized PβAE. The results displayed that the main peaks for the functional groups of the PβAE are located at 2965, 1681, and 1209 cm^−1^ for the C–H stretching band, C=O stretching, and C–O, respectively as the acrylate groups. Further, the distinctive signal at 1257 cm^−1^ for the C–N from the piperazine ring. These findings confirm the successful synthesis of PβAE. Meanwhile, the successful entrapment of TEINH in the polymeric matrix is illustrated by the appearance of the new weak overtones of the phenyl and the aromatic pyridine ring of the nicotinamide structure from 2000 to 1670 cm^−1^. Moreover, the new bands at 2925, 2100, 1660, and 1640 cm^−1^ are attributed to the C–H stretch (alkyl), N–N, N–H, and C=N stretching, consecutively, demonstrating the presence of the TEINH within the PβAE gel (Fig. 3). In addition, the generated signals between 2080 and 1672 cm^−1^ are confirmed the presence of the aromatic pyridine ring from APTAT. The N–N signal is at 2095 cm^−1^, the C=S stretching frequency has been found around 1202 cm^−1^ and the band of C=N is at 1645 cm^−1^ which proves the loading of APTAT into PβAE through Fig. 3. Further, the conformation of MOAPM entrapment is observed in Fig. 3, where the existence of the new entity peaks at 2450, 2085, 1650, and 1020 cm^−1^ for the S–H stretching, N–N, C=N, and S–H bending vibrations, respectively. The overtone peaks in the range from 2076 to 1673 cm^−1^ and the 1212 cm^−1^ of the C–O also indicate that the MOAPM physically interacted with the PβAE.

### 3.3. SEM analysis

**Fig. 4:**
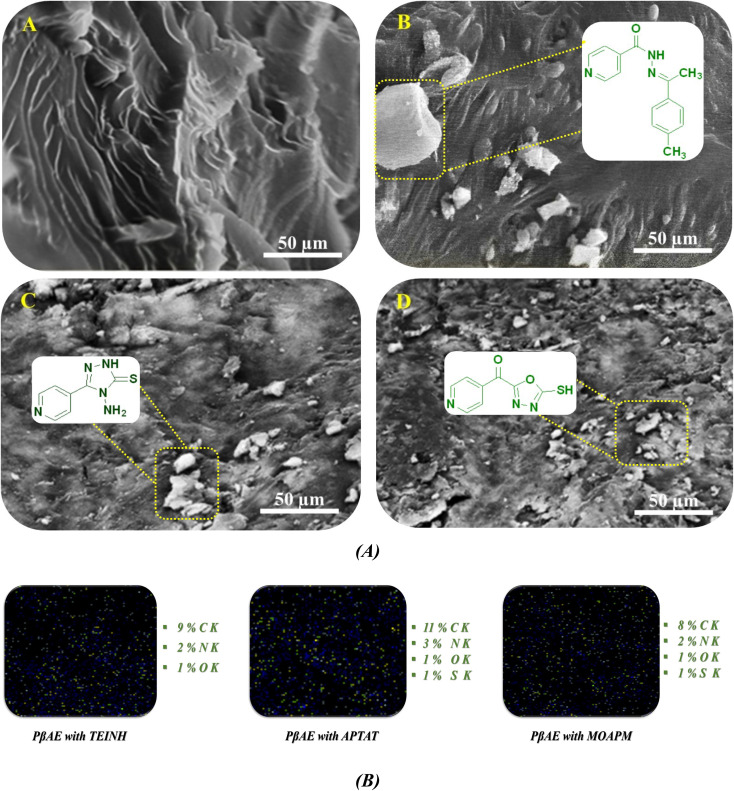
(A and B) SEM graphs of (A) pure PβAE and loaded PβAE with (B) TEINH, (C) APTAT, and (D) MOAPM and EDX mapping.

The scanning electron microscope is a useful tool for investigating the allocation and disseminating of drugs through the surface of the test specimen. The PβAE texture surface prior to and following bioactive compounds entrapment should be compared for meaningful information. Fig. 4 shows a micrograph of the prepared products' prepared surfaces. Fig. 4A clarifies the investigation of pure PβAE before loading with pharmaceutical active materials. It turns out that the surface texture of the polymer is homogenous, with smooth gradient strips and layers. The micrographs in Fig. 4B–D after the pharmaceutical materials have been loaded reveal that the polymer surface still has some homogeneity with bits and ridges embedded in it, with good bioactive compound dispersion and penetration through large patches of the polymer's surface texture.

### 3.4. Drug delivery investigation

#### 3.4.1. Swelling capability of PβAE

**Fig. 5:**
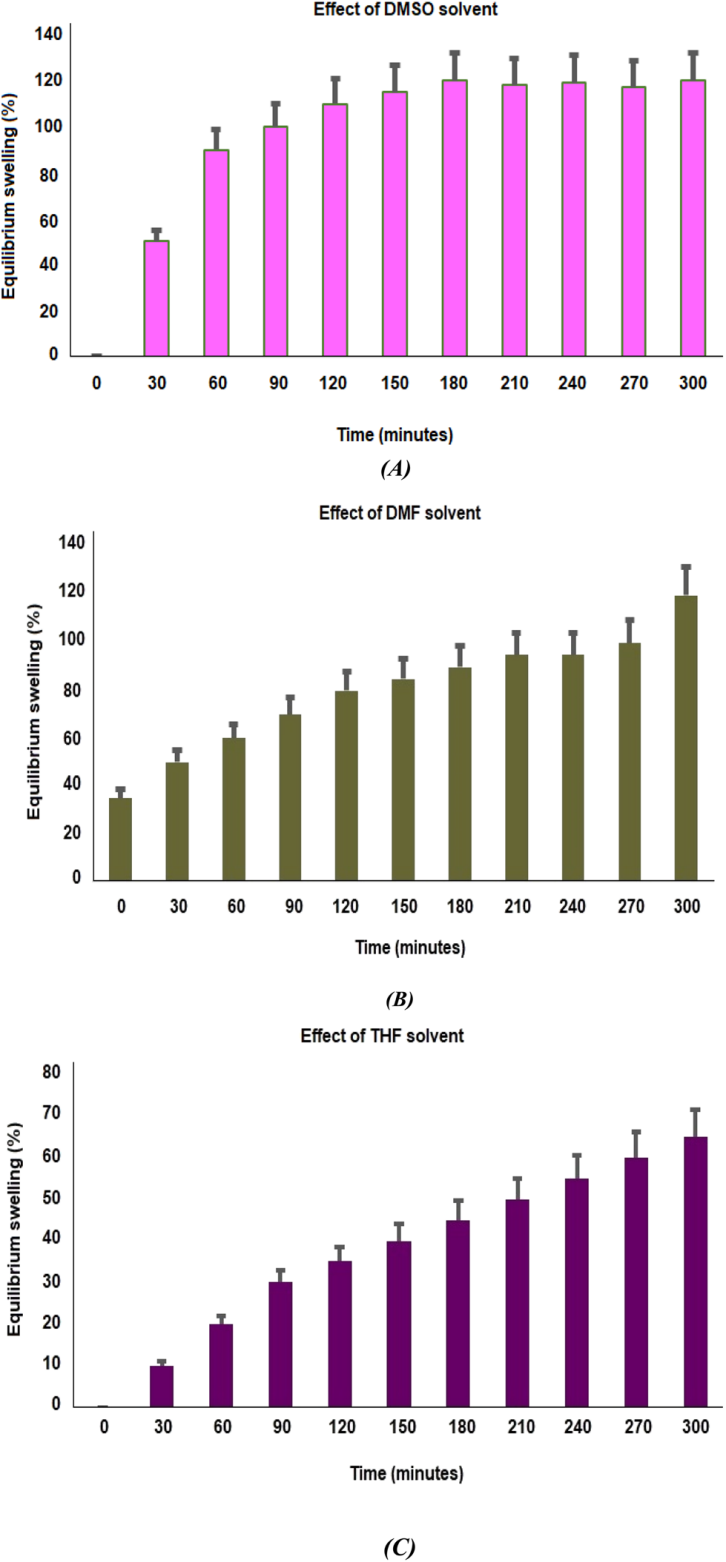
(A–C) Effect of different solvents on swelling response of PβAE *via* DMSO, DMF, and THF with Standard deviation analysis; respectively.

Because it greatly affects the way drugs are released, swelling behavioral patterns are a crucial characteristic for drug delivery systems. To investigate how different solvents interact with polymers, the swelling study of the PβAE matrix was carried out in DMSO, DMF, and THF at room temperature. According to Fig. 5, the maximum swelling is attained for all solvent systems in a little under 60 minutes. The maximal enlargement of the green synthesized PβAE was noticed in the cases of DMSO (alkaline media, pH 9–10), DMF (neutral media, pH 6.8–7.4), and THF (acidic media, pH 4–5), in roughly 5 hours. The factor of interaction system between polymer and solvent, which is a measure of the polymer's dissolution in the solvent, may be able to explain this dependency of expansion on the polymer–solvent system.^37^ These outcomes indicate that the eco-friendly PβAE-developed matrix is suitable as a carrier for pharmaceutical drugs with a target in a basic medium.

#### 3.4.2. Biodegradation of PβAE

**Fig. 6:**
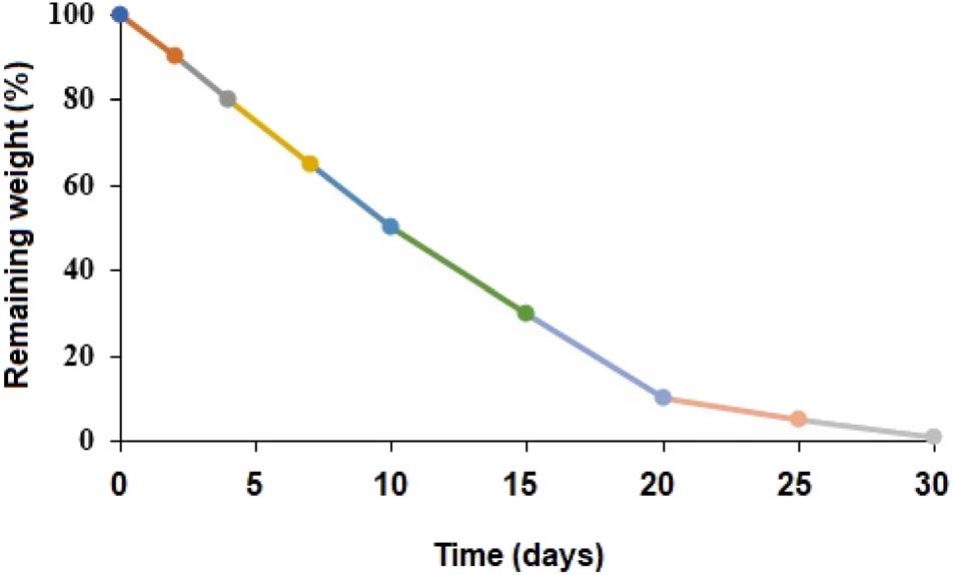
*In vitro* biodegradation of PβAE.

As demonstrated in Fig. 6, the weight reduction of the polymer throughout the duration of an incubation period in PBS (with a pH of 7.4) at 37 °C allowed for tracking the biodegradability of the PβAE matrix. *In vitro*, the PβAE degraded relatively quickly within the first 2 days, with a 10% mass loss. After 7 days from the beginning of the degradation study, the mass decreased by 35% from its initial mass. The remaining weight within 10 days was 50%, while the following 15 days the weight loss was 95%. Complete degradation through loss of all PβAE mass reduction was within 30 days from the study beginning. The data from biodegradation investigations in PBS (with a pH of 7.4) at 37 °C were consistent with a polymer that degraded hydrolytically as time passed. The relative simplicity of surface degradation *via* molecules of water was responsible for the faster deterioration profile that was comparable to other manufactured polyesters.^40^ It has been previously reported that PβAE degraded through the hydrolysis of ester groups in the crosslinks, resulting in lower molecular weight degradation products and kinetic chains of poly(β-amino acids) and diols.^41^

#### 3.4.3. Entrapment and controlled release of TEINH, APTAT, and MOAPM from PβAE

**Fig. 7:**
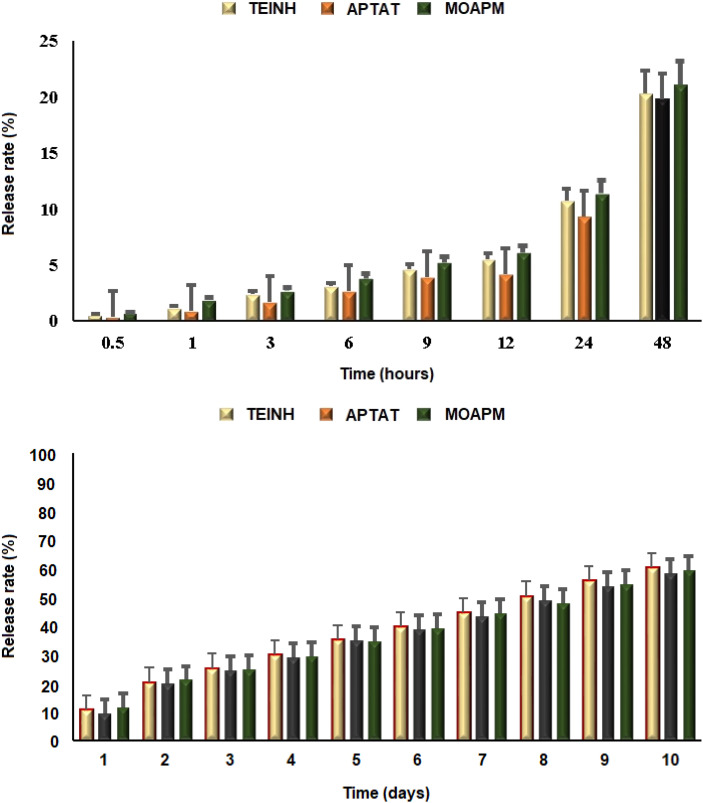
The release rate percentage of TEINH, APTAT, and MOAPM from PβAE.

This study evaluated the potential of PβAE for medication delivery through studying the *in vitro* release measurements of synthesized antitumor drugs from the polymeric carrier, achieving a 100% entrapment efficiency rate. The spectrophotometric release rates of TEINH, APTAT, and MOAPM from PβAE were determined under their optimum lambda values utilizing PBS (pH 7.4) at 230, 245, and 255 nm, respectively. According to the release curves in Fig. 7, the controlled release rate of the bioactive nicotinamide derivatives from the PβAE was achieved; after 2 and 10 days, only about 20% and 60% of the entrapped medicinal materials were released, respectively. These studies reveal that the PβAE is stable and effective in preventing drug leakage during medication administration. The active synthesized nicotinamide molecules are maintained trapped in the core of polymeric matrix because the tertiary amine of PβAE is not protonated at pH 7.4. However, upon incubation in PBS (with a pH of 7.4), the bioactive substances were slowly released in varying amounts.

## 4. Biological studies

### 4.1. Biological efficiency of antimicrobial strains

**Fig. 8:**
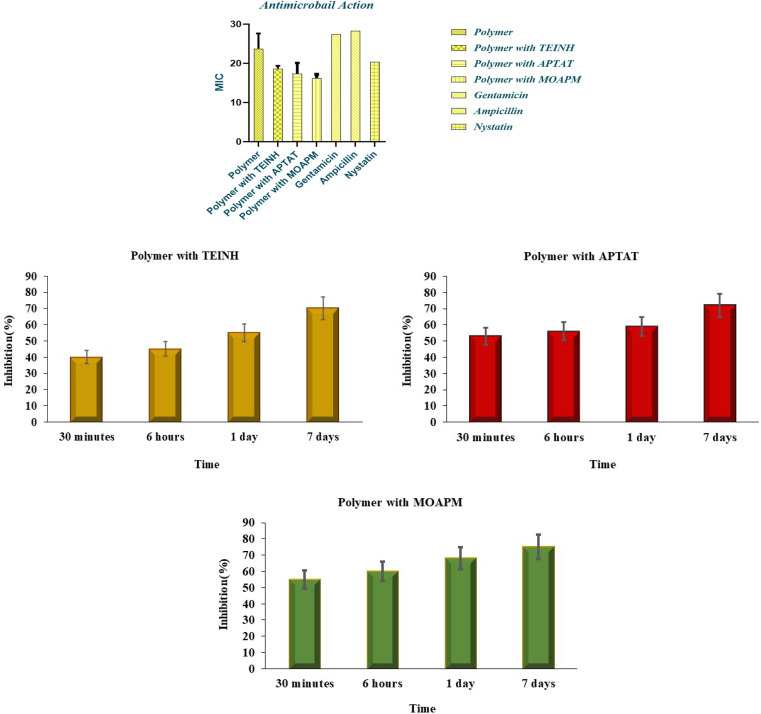
Inhibition zone diagrams with statistical of antimicrobial actions of free PβAE and PβAE loaded with TEINH, APTAT, and MOAPM.

Antimicrobial action of synthesized poly(β-amino ester) with TEINH, APTAT, and MOAPM was verified against inhibitory growth.^30^ for Gram-negative bacteria *Escherichia coli* (ATCC:10536),^42^ Gram-positive bacteria *Staphylococcus aureus* (ATCC:13565),^43^ Fungi (*Candida albicans* (ATCC:10231),^44^ and *Aspergillus niger* (ATCC:16404)^45^ as demonstrated in Table 2 and Fig. 8.^45^ The study, the participation of isonicotinic ring and its implications on the antimicrobial ability against Gentamicin, Ampicillin, and Nystatin.^46^ From the observations in Table 2 and Fig. 8, it was evident that the activity of compounds against *Escherichia coli* (ATCC:10536) exhibited less efficacy with poly(β-amino ester) (28.55 ± 0.5b), comparable to the standard drug Gentamicin (27 ± 0.5). However, an increase in activity was observed in the presence of isonicotinic derivatives, specifically TEINH, APTAT, and MOAPM (24.3 ± 0.3b, 23.8 ± 0.22b, and 21.4 ± 0.21b), respectively. This suggests a physical interaction occurring at the surface of the amino ester polymer, contributing to an enhanced inhibition zone of bacteria and increased activity.

**Table 2:** Antimicrobial action screened of free PβAE and PβAE loaded with TEINH, APTAT, and MOAPM with 2 mg per disc concentration associated with reference drug^a^

Sample microorganism	PβAE	PβAE with TEINH	PβAE with APTAT	PβAE with MOAPM	Standard antibiotic
**Gram-negative bacteria**					
					**Gentamicin**
*Escherichia coli* (ATCC:10536)	28.55 ± 0.5	24.3 ± 0.3	23.8 ± 0.22	21.4 ± 0.21	27 ± 0.5a

**Gram-positive bacteria**					
					**Ampicillin**
*Staphylococcus aureus* (ATCC:13565)	NA	22.5 ± 0.21	26.2 ± 0.21	24.4 ± 0.2	22 ± 0.1a

**Fungi**					
					**Nystatin**
	23.2 ± 0.1	20.12 ± 0.32	17.32 ± 0.23	19.2 ± 0.23	21 ± 0.5a

a Zone of inhibition is expressed in the form of mean ± standard deviation (mm), NA: no activity, well diameter (6 mm), 100 μl was tested, values that share the same letter at the same row are not significant, values that share different letters at the same row are significant.

In the case of *Staphylococcus aureus* (ATCC:13565), representative of Gram-negative bacteria, lower activity was observed with the polymer alone. The polymer combined with TEINH, APTAT, and MOAPM showed respective activity levels of (22.5 ± 0.21b, 26.2 ± 0.21b, and 24.4 ± 0.2b), comparable to the standard drug Ampicillin (22 ± 0.1a). Furthermore, the fungi exhibited the highest activity for all compounds, compared with Nystatin (21 ± 0.5a). Specifically, TEINH, APTAT, and MOAPM showed activity levels of (23.2 ± 0.1a, 20.12 ± 0.32b, 17.32 ± 0.23b, and 19.2 ± 0.23a), respectively. These results indicate that the activity of isonicotinic derivatives increased the inhibition zone, attributed to the presence of different functional groups such as NH, SH, and NH_2_. Additionally, the release of these drugs on the polymer surface occurred over different periods, ranging from 30 minutes to 7 days. Specifically, the release of the isonicotinic derivative (TEINH) from the polymer surface demonstrated an initial release of 40% of the heterocyclic, increasing to 45%, 55%, and 70% over time. APTAT showed a release of 72% at 7 days, while MOAPM exhibited a release of 75% at 7 days. The physical interaction between the polymer and these drugs, coupled with the presence of SH, NH, and NH_2_, facilitated their gradual release from the surface over time.^6,47,48^

### 4.2. Anti-proliferative activities and drug release

#### 4.2.1. Antitumor activity on MCF-7 breast cell line

**Fig. 9:**
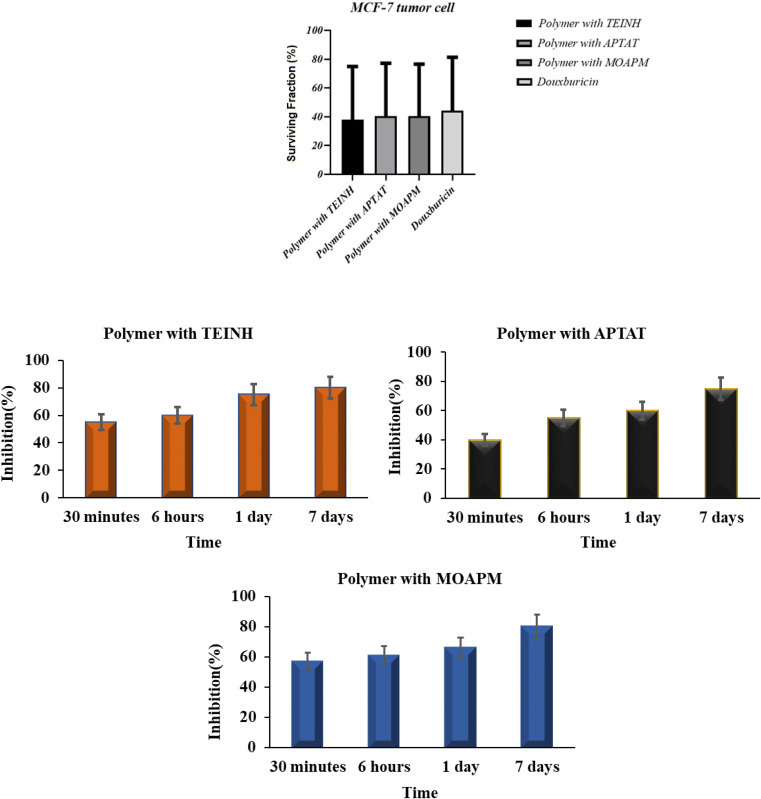
Antitumor activity and statistical of free PβAE and PβAE loaded with TEINH, APTAT, and MOAPM.

Anti-proliferative activity of the PβAE and PβAE with TEINH, APTAT, and MOAPM was evaluated *via* one human cancer cell line, namely, MCF-7 breast cancer using a pronounced sulforhodamine B (SRB) colorimetric test.^49,50^ Doxorubicin was used as a control cytotoxic drug in the investigations. The findings were represented as growth inhibiting concentration values (IC_50_), which indicate the material concentrations necessary to produce a 50% inhibition of cell division after 72 h of incubation compared to the untreated controls^51,52^ (Table 3 and Fig. 9). The results indicated that both PβAE and PβAE loaded with nicotinic heterocycles exhibited a significant to moderate inhibitory effect on the tested cancer cell type. Specifically, the breast tumor cell MCF-7 demonstrated heightened activity with the polymer combined with APTAT and MOAPM (IC_50_ = 6.321 ± 0.31 μg ml^−1^ and IC_50_ = 6.012 ± 0.27 μg ml^−1^, respectively), comparable to the effectiveness of doxorubicin (IC_50_ = 1.17 ± 0.07 μg ml^−1^). These promising outcomes were attributed to the presence of thiotriazole and oxadiazole rings attached to the polymer surface. Conversely, the polymer and TEINH-loaded PβAE exhibited lower activity (IC_50_ = 7.322 ± 0.23 μg ml^−1^ and 9.32.11 ± 0.53 μg ml^−1^), which could be attributed to the open chain structure in the polymer and TEINH's reliance solely on the activity of N=CH, resulting in decreased inhibition of antitumor activity. Additionally, the absence of active sites on the surface of PβAE contributed to reduced inhibition of tumor cell growth. Furthermore, the release of these isonicotinic derivatives from the PβAE surface spanned from 30 minutes to 7 days, reaching up to 80% after an extended period. This suggests that these nicotinic drugs demonstrated excellent stability over an extended duration when prepared and loaded onto the amino ester surface, highlighting their non-toxic nature on tumor cells.

**Table 3:** The study assessed the cytotoxic effects of PβAE and nicotinic heterocycles on the MCF-7 breast cell line *in vitro*

(MCF-7)	
Compound	IC_50_(μg ml^−1^)^a^
PβAE	9.32.11 ± 0.53
TEINH-loaded PβAE	7.322 ± 0.23
APTAT-loaded PβAE	6.321 ± 0.31
MOAPM-loaded PβAE	6.012 ± 0.27
Doxorubicin	1.17 ± 0.07

a IC_50_ values are the mean ± S.D. of three separate experiments.

### 4.3. Optimization of the synthesized materials

**Fig. 10:**
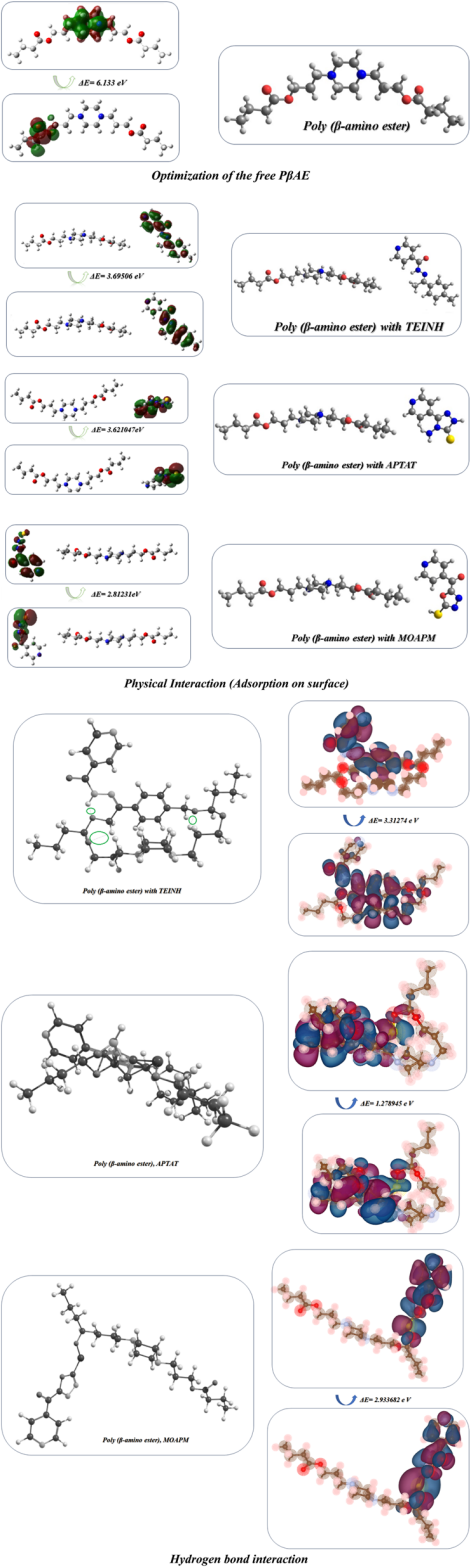
Optimization of physical and hydrogen bond interaction with FMO of free PβAE and PβAE loaded with TEINH, APTAT, and MOAPM.

In this study, Gaussian(09) was employed for the optimization of TEINH, APTAT, MOAPM, PβAE, and PβAE loaded with TEINH, APTAT, and MOAPM.^53–55^ through the use of the DFT/B3LYP/6-31(G) basis group, which concerned their physical properties, (*σ*) absolute softness,^56^ (*χ*) electronegativities,^54,57^ (Δ*N*_max_) electronic charge,^58^ (*η*) absolute hardness,^59^ (*ω*) global electrophilicity,^60^ (*S*) global softness,^26,58,60^ and (Pi) chemical potential,^61^ from the eqn (5)–(12) which were scheduled in Table 4 and Fig. 10.

5 Δ*E* = *E*_LUMO_ − *E*_HOMO_

6
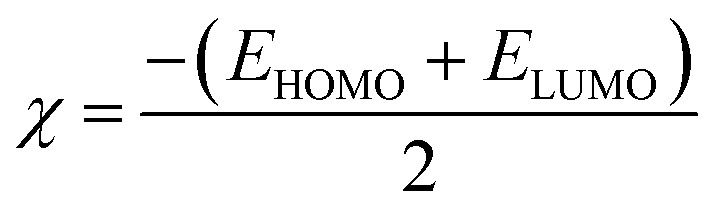
7
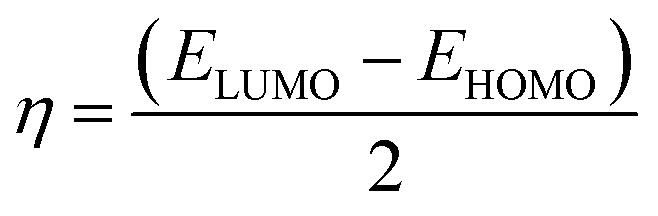

8 *σ* = 1/*η*

9 Pi = −*X*

10 *S* = 1/2*η*

11 *ω* = Pi2/2

12 Δ*N*_max_ = −Pi/*η*

**Table 4:** Physical descriptors of free PβAE, PβAE loaded with TEINH, APTAT, and MOAPM through DFT/B3LYP/6-31G(d) basis set

	PβAE	TEINH	PβAE with TEINH	APTAT	PβAE with APTAT	MOAPM	PβAE with MOAPM
*E*_T_ (au)	−1116.40	−840.61	−2403.28	−942.54	−2363.97	−619.73	−2552.37
*E*_HOMO_ (eV)	−5.95	−6.312	−5.88	−5.544	−5.86	−5.82	−5.58
*E*_LUMO_ (eV)	0.18	−2.48	−2.39	−1.93	−2.49	−1.98	−2.64
*E*_g_ (eV)	6.13	3.83	3.69	3.62	3.62	3.83	2.81
μ (D)	1.53	4.13	9.90	1.47	10.16	1.15	8.70
*χ* (eV)	2.88	4.39	4.14	3.738	4.17	3.91	4.11
*η* (eV)	3.06	1.92	1.74	1.809	1.68	1.918	1.47
*σ* (eV)	0.32	0.522	0.57	0.553	0.59	0.52	0.68
Pi (eV)	−2.88	−4.395	−4.14	−3.738	−4.18	0.521	−4.12
*S* (eV)	0.16	0.261	0.29	0.276	0.29	−3.905	0.34
*ω* (eV)	1.35	5.041	4.92	3.861	5.17	0.261	5.77
Δ*N*_max_	0.94	2.29	2.38	2.061	2.47	3.975	2.80

The optimization of the PβAE revealed a total energy of (−700557.24 kcal mol^−1^) and a band energy gap between its HOMO and LUMO of (6.13 eV) (141.42 kcal mol^−1^), indicating the high stability of PβAE. The electron distribution was found to be homogeneous, with the LUMO primarily located in the piperazine ring and the HOMO in the C=O of butanediol, contributing to enhanced stability. Additionally, the low dipole moment of PβAE (1.5333 Debye) suggests that the bonds within the polymer are nonpolar, indicating similar electronegativity across the structure.,^36,62–64^ which showed (2.88 eV) (66.55 kcal mol^−1^). In addition, (*η*) absolute hardness showed a high value with (3.066 eV) (70.7036 kcal mol^−1^) and it was good to show that the core of the PβAE is strong enough to load onto its surface. (*σ*) absolute softness was investigated (0.32 eV) (7.51 kcal mol^−1^), (*S*) global softness showed (0.16 eV) (3.75 kcal mol^−1^) explained these values. The surface of this polymer is smooth and can excrete the drug from its surface,^65^ as demonstrated in Fig. 10 and Table 4.

Furthermore, the optimization of PβAE in combination with the compounds TEINH, APTAT, and MOAPM revealed the physical interactions between them, as illustrated in Fig. 10. Firstly, when PβAE was combined with (*E*)-*N*′-(1-(*p*-tolyl)ethylidene)isonicotinohydrazide, the total energy was calculated as (−65396.517 eV) (−1508079.1321 kcal mol^−1^). This value was higher than that of the standalone PβAE, indicating interaction. The Δ*E* value, indicating the difference in energy levels, was 3.69 eV, lower than the polymer itself. Delocalization of electrons, particularly in the NH–N=C moiety of nicotinohydrazide, facilitated electron charge transfer, enhancing the compound's activity on the polymer surface. The increased dipole moment (9.91 Debye) suggested enhanced polarity of the C=N bond, promoting easy interaction and raising electronegativity (*χ*) to 4.14 eV. Despite the lower absolute hardness compared to the standard polymer (1.74), its smoother surface and increased softness (0.57) indicated enhanced reactivity due to the presence of nicotinic acid, NH, and N=CH, with a collision diameter of 16.191622 Å. Secondly, the physical adsorption of PβAE with 4-amino-5-(pyridin-4-yl)-2,4-dihydro-3*H*-1,2,4-triazole-3-thione (APTAT) resulted in an increased total energy of the polymer (−64326.928173 eV) (−1483413.8253 kcal mol^−1^). The difference in transition states of HOMO–LUMO was 3.621047 eV, and delocalization of electrons on the amino thio triazole indicated enhanced action on the polymer surface, increasing system polarity. The higher dipole moment (10.1653 Debye) added to the polarity, resulting in a collision diameter between them of 14.3878356 Å. Additionally, the adsorption of polymer with (5-mercapto-1,3,4-oxadiazol-2-yl)(pyridin-4-yl)methanone (MOAPM) showed the most stability on the polymer surface with a total energy of (−69453.59 eV) (−1601637.55 kcal mol^−1^).^15,66^ and the presence of oxadiazole ring, nicotinic, and SH got the stability of it and led to a minimum band gap energy of 2.81 eV, which can easily electron transfer than the polymer itself and its polarity, 8.71 Debye due to the presence of SH only in the compound, while the increase in compound 5 is due to the presence of two functional groups, NH_2_ and SH, which increase polarity, and the polymer 6 showed an electronic charge of 2.80 with a high value due to the delocalization of electrons in the system with collision diameter between them is 14.959789 Å.^63,66,67^ Additionally we make optimization of the PβAE, PβAE loaded with TEINH, APTAT, and MOAPM through hydrogen bond interaction and showed different band energy gap with Δ*E* = 3.31274 eV, Δ*E* = 1.278945 eV and Δ*E* = 2.933682 eV with more stability with TEINH and MOAPM, while it showed high reactivity with APTAT due two hydrogen bond interaction between NH_2_ and SH with the C=O of amino ester as displayed in Figure.10.^61,62,68^

Moreover, the binding and interaction energies of van der Waals force interactions between the poly(β-amino ester) and TEINH, APTAT, and MOAPM, and these investigation make individually for each compound and as showed in Tables 4 and 5 the difference of energy between optimized polymer and total energy of both compounds showed the least energy of poly(β-amino ester) with MOAPM than other compounds and it is confirmed our investigation and its due to the presence of oxadiazole ring, nicotinic, and SH which increase the van der Waals force, additionally the TEINH with amino ester showed least energy with −280035.83 kcal mol^−1^ and it is related on interaction of amino ester with the CH_3_ and NH=NH and make the difference between their energy to low value and give it stability. Finally, the APTAT showed the least energy with −191409.26 kcal mol^−1^ to showed interaction with NH of triazole, NH_2_ and SH, and the high value of van der Waals force interactions is due to presence of lone pair of electrons from NH, SH, and O which increase this force.

13 Δ*E*_interaction_ = *E*_optimized PβAE loaded_ − (*E*_PβAE_ + *E*_nicotinamide drugs_)

**Table 5:** Difference energy of interaction between PβAE and TEINH, APTAT, and MOAPM

	Electronic (*E*) (au)	Electronic (*E*) (kcal mol^−1^)
Δ*E*_interaction(TEINH)_	−446.27	−280035.83
Δ*E*_interaction(APTAT)_	−305.03	−191409.26
Δ*E*_interaction(MOAPM)_	−816.24	−512198.44

## 5. Conclusion

In the present research, we utilized microwave irradiation to produce poly(β-amino ester) through addition polymerization. This was accomplished by reacting 1,4-butane diol diacrylate with piperazine, resulting in a high yield of the corresponding poly(β-amino ester) which was analyzed using spectral analysis. Additionally, we investigated the adsorption of different heterocycles such as TEINH, APTAT, and MOAPM, which showed physical interaction with the polymer surface, as confirmed by FT-IR and SEM analysis. The PβAE matrix effectively prevents drug leakage during medication administration. With only 20% and 60% of entrapped medicinal materials released after 2 and 10 days, respectively, because of the tertiary amine's stability. The resulting PβAE and isonicotinic heterocycles exhibited antimicrobial and antitumor action on MCF-7 tumor cells. The release of these compounds from the surface of the PβAE took a long time, indicating their reactivity and excellent performance over extended periods. Moreover, the physical adsorption investigation revealed that the band energy gap of the PβAE entrapping TEINH, APTAT, and MOAPM in the HOMO–LUMO is smaller than that of the free PβAE. This suggests the reactivity of these heterocycles on the surface of the polymer and their ability to form stronger van der Waals binding interactions with their functional groups.

## Data availability

The authors are ready to provide data at request.

## Conflicts of interest

We have no conflict of interest with anyone.

## Supplementary Material
